# Efficacy of Gold Nanoparticles against Drug-Resistant Nosocomial Fungal Pathogens and Their Extracellular Enzymes: Resistance Profiling towards Established Antifungal Agents

**DOI:** 10.3390/nano12050814

**Published:** 2022-02-28

**Authors:** Abobakr Almansob, Ali H. Bahkali, Fuad Ameen

**Affiliations:** Department of Botany & Microbiology, College of Science, King Saud University, Riyadh 11451, Saudi Arabia; aalmansob@ksu.edu.sa (A.A.); abahkali@ksu.edu.sa (A.H.B.)

**Keywords:** *Mentha piperita*, nosocomial fungi, AuNPs, antifungal, extracellular enzymes

## Abstract

Drug resistance of filamentous fungi to the commonly used antifungal agents is a major concern in medicine. Therefore, an effective approach to treat several opportunistic fungal infections is the need of the hour. *Mentha piperita* is used in home remedies to treat different disorders. Isolates of fungi were taken from hospitals in Riyadh, Saudi Arabia, and identified using molecular tools. Amphotericin B, Voriconazole, and Micafungin were applied to screen the resistance of these isolates using both disc and broth microdilution techniques. An aqueous extract of *Mentha piperita* was utilized to synthesize AuNPs and the nanoparticles were characterized using UV-Vis, FTIR, TEM, EDAX, and XRD. The AuNPs were tested for antifungal activity against the nosocomial fungal pathogens and the activity of extracellular enzymes of such pathogens were analyzed after treatment with AuNPs. We conclude that AuNPs synthesized using *Mentha piperita* do not possess especially effective antifungal properties against multi-drug resistant Aspergillus species. Five out of eighteen isolates were inhibited by AuNPs. When inhibition was observed, significant alterations in the activity profile of extracellular enzymes of the nosocomial fungi were observed.

## 1. Introduction

*Aspergillus* is an omnipresent, filamentous, mycotoxigenic fungus classified into a group of pathogens termed environmental opportunistic pathogens (EOPs) [1]. It is notably recognized for causing nosocomial invasive aspergillosis, a rigorous health hazard among immune-compromised patients [2,3]. Although limited reports are available on nosocomial invasive aspergillosis, it is observed to be concomitant with the maintenance activities of buildings in and around hospitals with limited chances of incidence among common inhabitants [4].

Lower respiratory infections result in several deaths and pose a serious concern in countries with developing economies [5]. Invasive aspergillosis is a lower respiratory infection primarily of nosocomial origin [6,7]. Members of *Aspergillus* spp., such as *Aspergillus niger*, *Aspergillus flavus*, *Aspergillus fumigatus*, and *Aspergillus terreus* are established pathogens isolated in patients with invasive pulmonary aspergillosis [8]. Recent studies propose using metallic nanoparticles for the management of other lower respiratory infections like COVID-19 [9].

Nanotechnology has become an established field of research over the recent decade and the use of nanomaterials is pivotal for this upsurge in medicine and diagnostics [10,11,12,13]. Due to the multiple advantages they possess, biosynthesis using a bottom-up approach is recently preferred over chemical or physical methods [14,15]. In particular, biosynthesis using plant extracts is gaining importance compared to microbe-assisted synthesis, due to the added advantages of the large volume of capping agents, laborious expenditures associated with microbial cultures, and separation techniques along with the reduced extent of toxicity and time. In addition, the phytochemicals of plant extracts can stabilize the nanomaterials, leading to relatively higher stability [16,17,18]. In some cases, plant enzymes like bromelain and vegetable wastes or their extracts have been used to synthesize AuNPs [19,20,21]. Adding to these benefits, metallic nanoparticles, notably AuNPs, synthesized for applications in medicine are known to possess antifungal properties, particularly against pathogens capable of causing respiratory infections [22,23,24,25,26,27].

Although research continues to combat resistance to antifungal agents due to their extensive usage, fungi continue to improve the resistance mechanisms. Based on their location of action, antifungal agents are categorized into the following classes: polyenes, azoles, allylamines, flucytosine, and echinocandins [28,29]. Most antifungal agents target the formation and functioning of ergosterol, the significant constituent of the fungal cell wall, and inhibit the synthesis of macromolecules [30]. As antifungal resistance has turned out to be a critical issue, new agents to stand against infections caused by fungi have become the need of the hour.

Owing to this background, AuNPs were synthesized and used in this study as antifungal agents against clinical pathogens responsible for nosocomial infections. In addition, this is the first-ever international report with regard to a few aspects: 1. On testing the efficacy of AuNPs on filamentous fungi, especially against multi-drug resistant *Aspergillus* species. 2. In evaluating the antifungal activity of AuNPs by both disc diffusion and broth microdilution methods for applications in nanomedicine against filamentous fungi. 3. In comparing the antifungal effects of AuNPs with existing standard drugs.

## 2. Materials and Method

### 2.1. Collection and Identification of Fungal Pathogens

#### 2.1.1. Sample Collection and Ethical Clearance

Blood samples were obtained from patients with aspergillosis in the microbiology departments of both King Khalid University Hospital and Regional Laboratory and Blood Bank of King Saud Medical City hospital over a period of 1 month. The fungal isolates were collected from the departments according to the guidance of the Ministry of Health-Kingdom of Saudi Arabia (MOH-KSA) and after the acceptance of the Institutional Review Board of King Khalid University Hospital. The isolates were collected, preserved in sterile bags, and transported to the laboratory under the most ideal conditions possible.

#### 2.1.2. Retrieval and Identification of Fungi

Eighteen isolates obtained from the source departments were revived, sub-cultured on Sabouraud’s agar (Oxoid Ltd., Basingstoke, UK), and incubated for 5 days at 28 °C. The pure cultures were stored in glycerol at −80 °C for further analysis. Mat morphology and microscopy were used for primary identification of the isolates. However, precise identification of the isolates was done on the basis of 18S rRNA sequencing and a comparison of homology with the sequences of the existing genotypes available in databanks.

#### 2.1.3. Screening for Antifungal Resistance

The fungal isolates were screened to establish the resistance to key antifungal agents: Amphotericin B (Cayman, Ann Arbor, MI, USA), Voriconazole (TCI, Tochigi, Japan), and Micafungin (Sigma-Aldrich, St. Louis, MO, USA). The Clinical and Laboratory Standards Institute (CLSI) M38-A standards were used as a reference to compare the minimum inhibitory concentration (MIC) obtained by Kirby–Bauer disc diffusion using modified the Espinel-Ingroff et al. technique [31,32,33]. Discs impregnated with DMSO were used as a control. The reference for each standard drug was: 1. Amphotericin B (10 µg); reporting resistance (R) when ZI ≤ 12 mm, Intermediate (I): ZI = 13–14 mm, and Susceptible (S): ZI ≥ 15 mm. 2. Voriconazole (10 µg); resistance (R) when: ZI ≤ 25 mm, Intermediate (I): ZI = 26–27 mm, and Susceptible (S): ZI ≥ 28 mm. 3. Reporting resistance to Micafungin was done resembling caspofungin (5 µg) applied in the Espinel-Ingroff et al. technique, resistance (R) when ZI ≤ 13 mm, Intermediate (I): ZI = 14–16 mm, and Susceptible (S): ZI ≥ 17 mm.

### 2.2. Synthesis and Characterization of AuNPs

#### 2.2.1. Preparation of the Plant Extract

M. piperita leaves were collected and identified by Government Botanical Survey and the voucher specimen was stored. The plant leaves were dried in an incubator (Memmert GmbH, Burladingen, Germany) for 7 days at 25 °C. The dried leaves were ground (ALSAIF ELEC grinder, 90,582) and washed thoroughly with double distilled water in order to remove any surface contaminants. The decoction extraction method was applied for the preparation of the aqueous extract. The extract was prepared in a specific 1:16 ratio (plant to solvent). A total of 10 gms of the powdered material was dissolved in 160 mL of double-distilled water. Overall, the concentration of extract was 0.06 g/mL (62.5 mg/mL or 62,500 ppm). The extracts were transferred to an incubator shaker (Gallenkamp, Gillingham, UK) and kept overnight at 25 °C. Later, filtration of the extract was achieved in two stages; with sterile large 124 cm filter papers (Whatman type No. 1, Gillingham, UK) and Acrodisc Syringe Filters (0.45 µm pore size/ Pall, New York, NY, USA) to eliminate any possible contamination.

#### 2.2.2. Synthesis of AuNPs

The aqueous plant extract was blended with 1 mM HAuCl_4_·3H_2_O (abcr GmbH, Burladingen, Germany) prepared under aqueous conditions in ratios of 1:1, 1:5, 1:10, and 1:15 and incubated at room temperature until a visible color change is observed. The control sample was prepared using the same test procedures, ratio, and conditions. Yet, sterilized distilled water was used in place of HAuCl_4_·3H_2_O. The purple solution obtained after incubation was centrifuged at higher centrifugal speeds (MiniSpin^®^, Eppendorf AG, Burladingen, Germany). The pellet was suspended in Milli-Q water and dried in an incubator for 24 hrs at 48–50 °C. Stocks of AuNPs were prepared at a concentration of 1000 ppm and serial dilutions of this stock were performed to obtain working solutions with concentrations of 200 ppm, 100 ppm, 50 ppm, and 25 ppm.

#### 2.2.3. Characterization of AuNPs

The biosynthesized AuNPs were initially characterized using a UV-visible spectrophotometer (UV-Vis, JASCO, New York, NY, USA). Possible encapsulates were identified using Fourier-transform infrared spectroscopy (FTIR, PerkinElmer Spectrum 100, New York, NY, USA). Elemental analysis was performed using energy-dispersive X-ray spectroscopy (EDX, JEOL, Tokyo, Japan). Morphology was observed using a transmission electron microscope (TEM, JEM 1011, Akishima, Tokyo, Japan). The crystalline nature was studied using X-ray diffraction (XRD, Rigaku Ultima IV, New York, NY, USA).

### 2.3. Antifungal Activity of AuNPs

Varying concentrations of AuNPs (25, 50, 100, 200, and 1000 ppm) were prepared and 10 µL of the solutions were loaded onto sterile discs. The antifungal activity was measured as a zone of inhibition in mm (mean ± SD). The broth microdilution technique was adopted following the CLSI M38-A protocol as a reference method to screen the accuracy and sensitivity of the disc diffusion method.

### 2.4. Effect of AuNPs on Extracellular Fungal Enzymes

The *A. flavus* (AM11) strain was used to test the influence of AuNPs on fungal metabolism represented by the enzymatic activity of the fungi. The profiling of nineteen extracellular enzymes was performed through API-Zym (Biomeruex, Craponne, France) strips test. The protocol was based on the standard method adopted by Pietrzak et al. [34], with slight modifications. The experiment was done by assigning two groups; test and control groups. In the test group, fungal inoculant (spore’s suspension: equivalent to 0.5 McFarland) in media (30 mL malt extract broth; MEB) was treated with AuNPs (1 mL of AuNPs for determining the minimum effective concentration—MEC) whereas, the control group was the same as the test group but without the AuNPs.

After treatment with AuNPs, the media was incubated for 7 days at 25 °C in 100 mL flasks. Subsequently, the fungal biomass was filtered through filter paper (Whatman type 1, Gillingham, UK). The filtrates were quantified for enzyme activity according to the standard API-Zym protocol depending on an increase in the intensity of color among the test sample in comparison to the control under identical conditions. A 0–5 score system was used, in which 0 determines no activity, while 5 determines maximum liberation of the hydrolyzed substrate. The concentration of enzymes was directly proportional to the intensity of the color. According to the scores 0, 1, 2, 3, 4, and 5, the enzyme activity will be 0, 5, 10, 20, 30, and ≥40 nmol, respectively.

### 2.5. Statistical Analysis

All experiments were performed in triplicate and represented as mean ± SD. Two-way ANOVA was applied and *p* < 0.05 was considered to be significant.

## 3. Results and Discussion

### 3.1. Identification of the Fungal Pathogens

**Figure 1:**
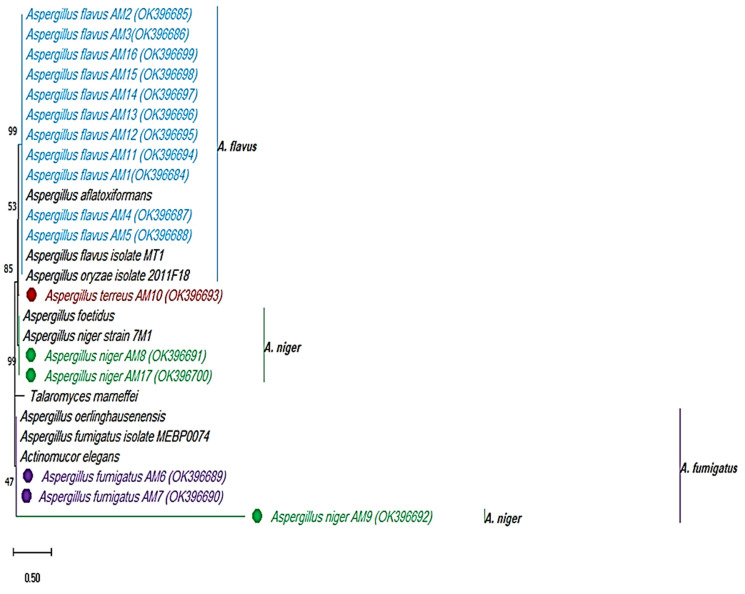
Identification of the clinical fungal pathogens by 18S rRNA sequencing. Maximum likelihood tree method and Tamura 3-parameter mode, 1000 bootstrapping by Mega X software.

Eighteen members of *Aspergillus* spp., such as *Aspergillus niger*, *Aspergillus flavus*, *Aspergillus fumigatus*, and *Aspergillus terreus* were identified in total. Seventeen isolates were identified by 18S rRNA sequencing. Each identified isolate was given an accession number and stored in NCBI. The other isolate termed *Aspergillus terreus* 8 was identified by mat morphology and microscopic observations. However, the identification of the isolates by use of molecular tools and phylogeny was assisted by the maximum likelihood tree method and bootstrapping by Molecular Evolutionary Genetic Analysis (Mega X) software [35] (Figure 1 and Table 1).

### 3.2. Screening for Fungal Resistance to Standard Antifungals

Considering the 300 pathogenic fungi of *Aspergillus* spp., *A. fumigatus*, *A. flavus*, *A. niger*, and *A. terreus* are considered significant in causing infections amongst immunocompromised patients [36,37]. These strains were isolated in patients with invasive aspergillosis of nosocomial origin, as per this study. Although infections caused by these pathogens are dependent on topographical and climatic conditions of countries, *A. flavus* is the most prevalent (61.1%) eukaryotic microbe in countries with hot and arid climatic conditions such as Saudi Arabia [38,39].

Among the eighteen tested strains in the disc method, seventeen strains (94.4%) were resistant (except *A. flavus* (AM15), which was sensitive (5.6%)) to Amphotericin B. *A. fumigatus* (AM6) was the most sensitive strain to this strong antifungal agent (Table 2). All tested strains were Voriconazole resistant (100%). In particular, *A. terreus* 8 was the most resistant (Table 3). Similar observations were made for Micafungin (Table 4). According to the standard broth microdilution technique, the susceptibility of fungal strains to Amphotericin B, Voriconazole, and Micafungin are shown in Table 5, Table 6 and Table 7. Owing to the CLSI method, all strains (18) were resistant to Amphotericin B, especially *A. terreus* (AM10), *A. flavus* (AM11), *A. flavus* (AM3), and *A. terreus* 8 with MIC >16 µg/mL. With respect to Voriconazole, most isolates were resistant, (72.2% (13)) especially *A. flavus* (AM4), *A. flavus* (AM14,) and *A. flavus* (AM15), whereas intermediate and high sensitivity profiles to Voriconazole were 16.7% (3) and 11.1% (2), respectively. Micafungin was not effective against most fungi tested 94.4% (17).

Overall, a broad resistance was observed and 55.6% of these resistant fungi opposed the antifungal activity as witnessed through the MIC ≥ 16 µg/mL of Amphotericin B. Furthermore, the resistance recorded with the other two antifungal agents was MIC ≥ 16 µg/mL in all resistant fungi. This grave matter of concern was documented to be higher than the expected and noticed values of other similar studies conducted either with polyene or azole antifungals [40,41,42]. Adding to this, the significant resistance recorded among *Aspergillus* spp. can either be intrinsic or of an acquired type. The latter type can occur as an outcome of the long-term habit of using antifungals for chronic aspergillosis, especially against *A. fumigatus*, *A. flavus*, or *A. terreus* [43]. Although Micafungin and Voriconazole have emerged recently as alternatives for the management of invasive aspergillosis, the resistance observed in this study was in contrast to the reports that support the susceptibility of fungi to these two agents [44,45,46].

### 3.3. Characterization of AuNPs

**Figure 2:**
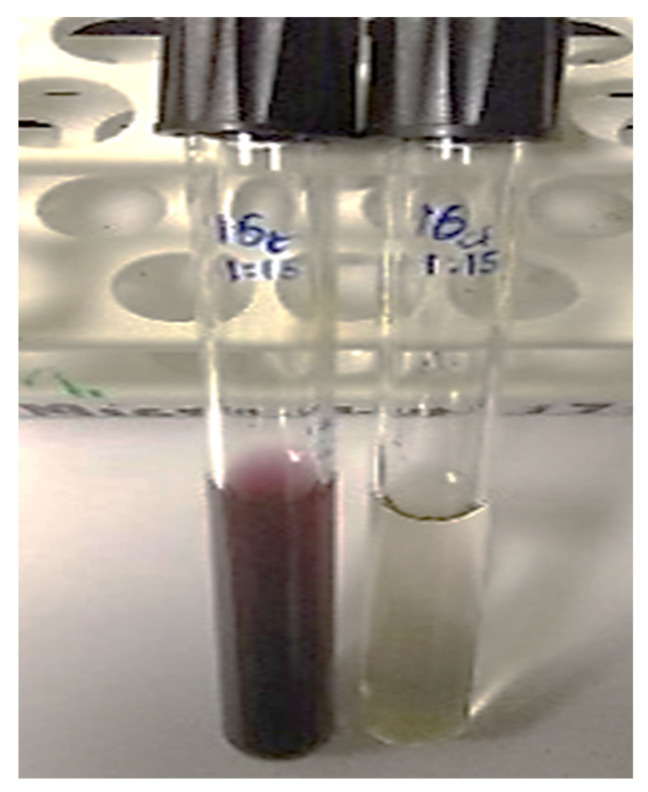
Initial confirmation of synthesis of AuNPs by a visible color change.

**Figure 3:**
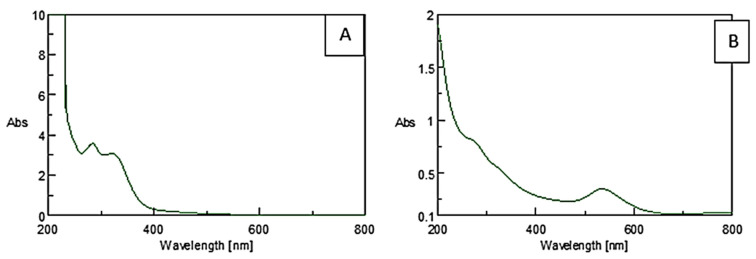
UV-Vis spectra of (**A**) plant extract and (**B**) AuNPs synthesized using *M. piperita*.

The visible color change to purple after incubating the precursor HAuCl_4_·3H_2_O with the plant extract is an initial confirmation for the reducing abilities of *M. piperita* (Figure 2) [47]. Surface plasmon resonance (SPR) is a band that occurs on the surface of metallic nanomaterials. Noticeable peaks in the range of 530 to 540 nm correspond to the SPR of AuNPs [48,49,50]. UV-Vis analysis indicates the formation of AuNPs by the plant extract as per the characteristic wavelength observed around 530 nm (Figure 3).

**Figure 4:**
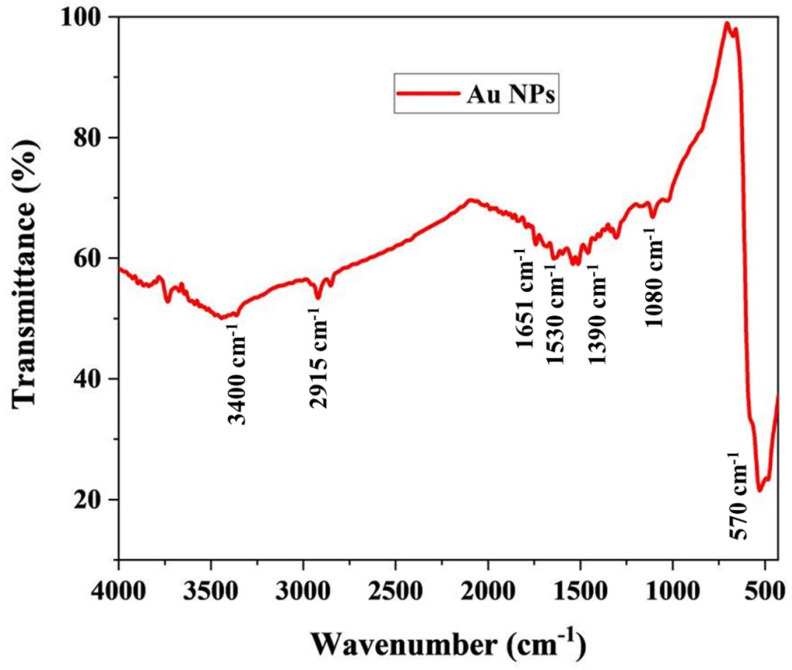
FTIR analysis of the hydrosol.

To further confirm the synthesis of AuNPs by *M. piperita* extract, FTIR was adopted (Figure 4). The FTIR peaks obtained in this study are supported well by previous reports which indicate the possible reducing and encapsulating agents on the AuNPs [51,52]. Extracts from plants of the genus *Mentha* rich in phenolic acids and essential oils are known to possess antifungal properties and are used as food preservatives [53,54]. According to a widely accepted hypothesis, a series of antioxidants, enzymes, and phenolics present in a plant extract can reduce the cations of gold to zerovalent gold. Consequently, the assemblage of gold atoms leads to the formation of AuNPs [55]. Phenylpropenes such as Apiol and Isoeugenol, Terpenoids such as Spathulenol, Ledene, α-Guaiene, and Pinene, and cyclohexanones such as Menthone are the active compounds of *M. piperita* extract. These compounds might act as reducing and stabilizing agents for the AuNPs [56].

The AuNPs synthesized by the *M. piperita* extract formed strong prominent bands at 510, 1080, 1390, 1530, 1651, 2915, and 3400 cm^−1^. The bands at 3400 cm^−1^ and 2915 cm^−1^ suggest the presence of stretching vibrations (O–H), and aldehydic C–H stretching, respectively. Again, their corresponding N–H bending vibration was seen at 1651 cm^−1^ and 1530 cm^−1^, respectively. In addition, the weak bands at 1390 cm^−1^ and 1080 cm^−1^ were assigned to C–N stretching vibrations of aromatic and aliphatic amines. The C–N stretching vibrations of aromatic and aliphatic amines were obtained at 1390 cm^−1^ and 1080 cm^−1^. The band observed at 570 cm^−1^ belongs to C–Br stretching vibrations which are consistent with the previous study [57].

**Figure 5:**
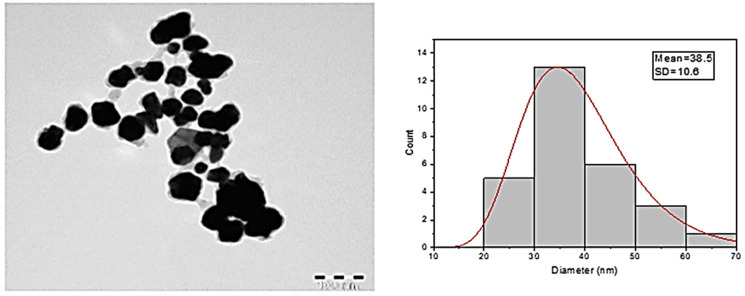
TEM images and diameter distribution of the AuNPs.

**Figure 6:**
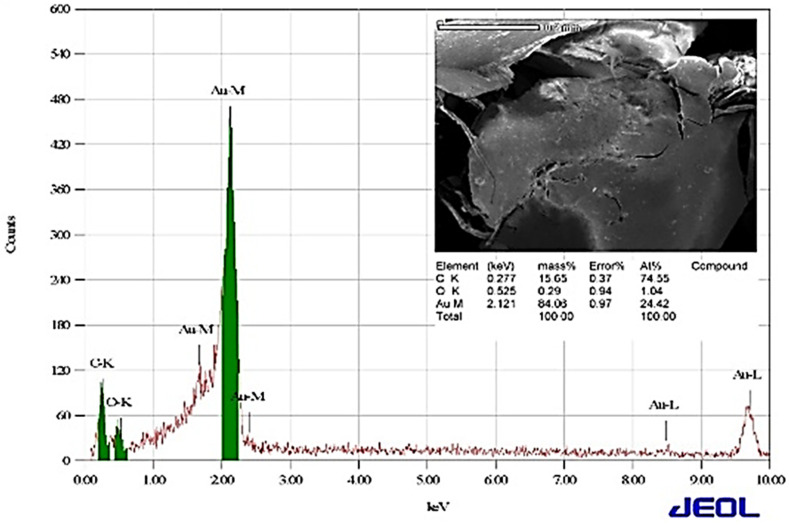
Analysis of elemental composition by EDAX.

**Figure 7:**
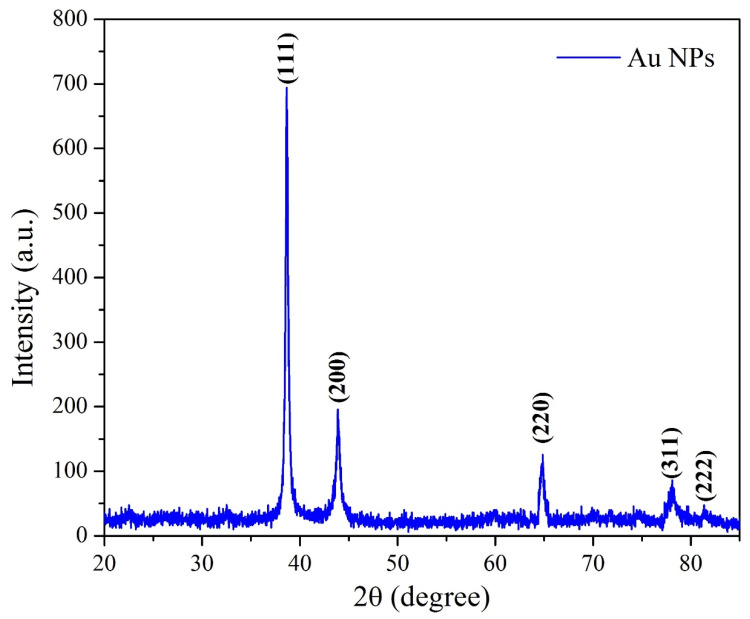
XRD pattern of the synthesized AuNPs.

After the possible encapsulates were identified, the morphology of AuNPs was studied using TEM. TEM is a widely applied technique to study the morphology of nanomaterials at various magnifications [58]. According to this technique, the synthesized AuNPs were predominantly spherical in shape (Figure 5). ImageJ software (1.8.0) predicted the size of the nanomaterials to be in the range of 38.5 ± 10.6 nm. After the morphology was studied, elemental mapping was performed by EDAX to determine the purity of the AuNPs. The composition of C (15.65%), O (0.29%), and Au (84.06%) indicates that the synthesized AuNPs were predominantly metallic gold forms. A strong and distinctive peak for AuNPs at 2.1 KeV was observed (Figure 6) [59]. After the morphology and elemental analyses were performed, the crystalline nature of the AuNPs was determined using XRD (Figure 7). The XRD peaks i.e., (111), (200), (220), (311), and (222) observed at their respective 2*θ* values, confirm the formation of face-centered cubic (fcc) structure of metallic gold which matched with the JCPDS No. 04-0784 [60,61]. The peak corresponding to the (111) plane is more intense than the other planes confirming that the plane (111) is the predominant orientation. The lattice constant (*a*) of AuNPs is calculated using the following formula (Equation (1)):

(1) $$\frac{1}{d^{2}}=\frac{h^{2}+k^{2}+l^{2}}{a^{2}}$$

where interplanar spacing (*d*), can be calculated by using Bragg’s law (2*d sinθ* = *λ*) and (*hkl*) are the Miller indices of the diffraction planes. The highest intensity diffraction peak belonging to the (111) plane is selected for calculation of the lattice parameter i.e., found to be 4.077 Å. The average crystallite size (*D*) was calculated from the XRD analysis using the Debye–Scherrer formula, given by Equation (2),

(2) $$D=\frac{0.9\lambda }{\beta cos\theta }$$

where *λ* is the wavelength of the X-ray radiation, *β* is the FWHM of the diffracted peaks, and *θ* is the glancing angle. The value of *D* is calculated to be ~24 nm which is concomitant with the result obtained from TEM analysis.

### 3.4. Antifungal Activity of the AuNPs

A marked antifungal activity of AuNPs against five out of 18 *Aspergillus* isolates was observed. Three out of eleven (28%) *A. flavus* isolates (AM2, AM11, and AM15) and both *A. terreus* isolates (AM10 and 8) were inhibited (Table 8). The inhibition zones in the highest AuNP concentration (1000 ppm) varied between 6.3 mm for *A.* *terreus* and 9.3 mm against *A. flavus* (AM2). The rest of the 18 isolates were not inhibited by AuNPs, showing 0 mm inhibition zones. The broth microdilution method showed the inhibition of AuNPs for all five isolates found susceptible in the disc method. The five isolates were remarkably inhibited by the highest AuNP concentration while *A. flavus* (AM2) was inhibited already in the 200 ppm concentration (Table 9). MIC was 1000 ppm for A. flavus (AM15) while MEC was 25 ppm for (AM2) (Table 9).

The AuNPs synthesized were not especially efficient against Aspergillus isolates. Only five out of eighteen isolates were inhibited by AuNPs. At the same time, the isolates were mostly resistant against the commercial drugs tested and needed the highest concentration to be inhibited. The relatively low inhibition efficiency of AuNPs is no surprise because it has been reported several times previously. AuNPs synthesized using the seed extract of Abelmoschus esculentus were not especially efficient against *A. flavus* and *A. niger* while they inhibited *Candida albicans* remarkably [62]. Elsewhere, AuNPs showed high activity against several Candida species [63]. The fungi Agaricus bisporus mediated AuNPs showed high antifungal activity against *A. flavus* but not against *A. terreus* [64]. When different synthesis and purification processes of the AuNPs were compared, the porification was observed to affect the efficiency of AuNPs against *C. albicans* [65]. Furthermore, AuNPs have been shown to inhibit *C. albicans* and *Sacharomyces cerevesiae* less than AgNPs [66]. AuNPs showed almost no activity against *C. albicans*, *C. tropicalis*, and *Fusarium oxysprorum*, while they were inhibited by NPs of Ag, Zn, and Cu synthesized using *A. kambarensis* extract [67]. Several other studies on varying and many times low efficiency against fungi have been published [55,68,69]. However, in some studies, high antifungal activity against *A. niger*, *A. flavus*, and *A. fumigatus* was also reported [70,71].

When thinking about the resistance against commercial drugs, the isolates that were inhibited by AuNPs behaved in various ways. For instance, *A. flavus* AM 15 needed the max concentration of 16 µg/mL Amphotericin B to be inhibited totally according to the microdilution method (Table 5). AM15 was inhibited to score 3 (out of 4) with 2 ug/mL concentration being relatively resistant. A. flavus AM11 was inhibited to score 3 only in the highest concentration. *Aspergillus terreus* AM10 was also resistant against Amphotericin B having a score of 3 at the highest concentration while a score of 4 up to 8 µg/mL concentration. *Aspergillus terreus* 8 was inhibited to score 2 only in the highest concentration. The least resistant isolate was *A. flavus* AM2 that was inhibited to score 3 already in the lowest concentration (1 µg/mL) and totally in 8 µg/mL.

### 3.5. Effect of AuNPs on the Activity of Fungal Extracellular Enzymes

The mechanism of antifungal activity of metallic nanoparticles has been attributed to several factors at molecular or physiological levels at membrane levels such as cell wall degradation or changes in the activity of extracellular enzymes [72,73]. Hence, we further tested the activity of extracellular enzymes before and after treatment with AuNPs.

Members of the *Aspergillus* genus are known to produce extracellular enzymes such as amylase, protease, deoxyribonuclease (DNase), lipase, elastase, and keratinase for growth, reproduction, and survival inside the host [74,75]. These extracellular enzymes are responsible for the formation of the extracellular matrix which can help in fungal evasion of killing by neutrophils and leading to a blockade in the production of reactive oxygen species. The components of the extracellular matrix of these pathogenic fungi (e.g., polysaccharides) are positively regulated by extracellular enzymes and can protect fungi from attacks by the host immune system [76]. Therefore, the extracellular components are outstanding targets for antifungal therapy [77].

With this background, the determination of the activity of extracellular enzymes after treatment with the AuNPs can be used as a tool to predict the possible survival mechanism. Hence, in this study, the effect of AuNPs on the extracellular enzymes was tested. *Aspergillus flavus* was selected in this report due to its high cytotoxic feature and its significant role in respiratory tract infections (invasive aspergillosis) and resultant complications in immunocompromised patients [78].

**Figure 8:**
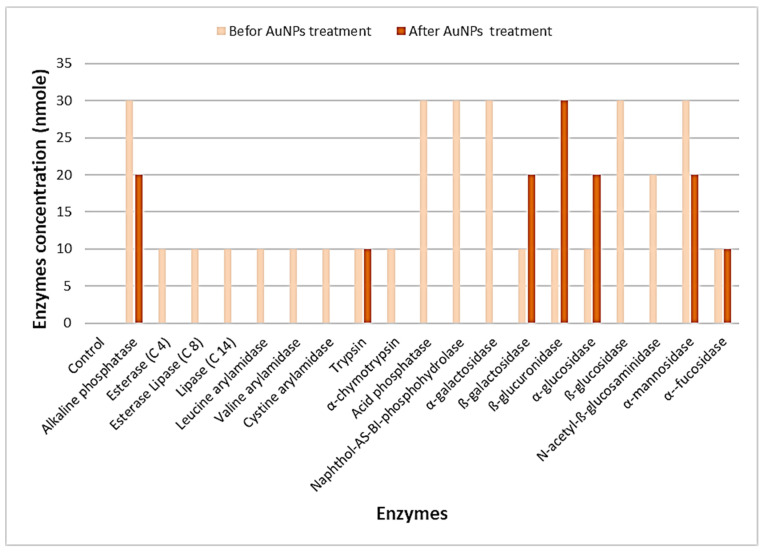
Effects of AuNPs on the activity of extracellular enzymes of *A. flavus* (AM11).

Profiling of extracellular enzymes produced by *A. flavus* (AM11, the most susceptible fungi) before treatment with AuNPs indicated that enzymes such as alkaline phosphatase, acid phosphatase, Naphthol-AS-BI-phosphohydrolase, α-galactosidase, ß-glucosidase, and α-mannosidase possessed higher activity (20 ≥ 40 nmoles among 36.8% of enzymes). Low to moderate amounts of activity (5–10 nmoles among 63.2% of the enzymes) were noted predominantly among other enzymes identified. After treatment with AuNPs, the activities of enzymes such as acid phosphatase, Naphthol-AS-BI-phosphohydrolase, α-galactosidase, and ß-glucosidase decreased (82.4% of changes in the altered profile of enzymes). However, the activity profiles of enzymes such as ß-glucuronidase increased. The outcomes indicate that the activity of fungal extracellular enzymes diminished significantly after treatment with AuNPs (Figure 8).

## 4. Conclusions

*Mentha piperita* represented a strong source for the synthesis and formation of AuNPs. The synthesized AuNPs were characterized structurally and morphologically. Further, the possible encapsulates were identified. In addition, this is the first-ever international report with regard to a few aspects related to antifungal effects, as mentioned at the end of the introduction section. Our findings related to the resistance pattern of these nosocomial isolates to common antifungals may indicate the emergence of serious acquired resistance to such agents, which might have arisen from the misuse of antifungals. This problem needs to be highlighted for proper maintenance of fungal infections in the future. Additionally, there are limited studies published in Saudi Arabia focusing on resistant *Aspergillus* and the worsening threat. Hence, this study would be effective for the use of nanomedicine in the management of nosocomial fungal infections of the respiratory tract. To conclude, the synthesized AuNPs were effective against five isolates of *Aspergillus* species that can cause invasive aspergillosis. Further studies are warranted to elucidate the interactions of these nanomaterials with the eukaryotic fungi at molecular levels.

## Figures and Tables

**Table 1:** List of fungi isolated and their NCBI accession numbers.

NO.	Strain Code	Fungi (Similarity: 100%)	NCBI Accession No.
1	(AM1)	*Aspergillus flavus*	OK396684
2	(AM2)	*Aspergillus flavus*	OK396685
3	(AM3)	*Aspergillus flavus*	OK396686
4	(AM4)	*Aspergillus flavus*	OK396687
5	(AM5)	*Aspergillus flavus*	OK396688
6	(AM6)	*Aspergillus fumigatus*	OK396689
7	(AM7)	*Aspergillus fumigatus*	OK396690
8	(AM8)	*Aspergillus niger*	OK396691
9	(AM9)	*Aspergillus niger*	OK396692
10	(AM10)	*Aspergillus terreus*	OK396693
11	(AM11)	*Aspergillus flavus*	OK396694
12	(AM12)	*Aspergillus flavus*	OK396695
13	(AM13)	*Aspergillus flavus*	OK396696
14	8	*Aspergillus terreus* 8	-
15	(AM14)	*Aspergillus flavus*	OK396697
16	(AM15)	*Aspergillus flavus*	OK396698
17	(AM16)	*Aspergillus flavus*	OK396699
18	(AM17)	*Aspergillus niger*	OK396700

**Table 2:** Susceptibility of fungal species to different concentrations of Amphotericin B (disc method).

No.	Isolate	Inhibition Zone (mm)			
		1 µg/mL	2 µg/mL	4 µg/mL	10 µg/mL
1	*A. flavus (AM1)*	2 ± 3.5	6.7 ± 0.6	7.7 ± 0.6	9.39 ± 0.6 ^(R)^
		9	9	9	9
2	*A. flavus (AM2)*	2± 3.5	7.7 ± 0.6	8.7 ± 0.6	9.7 ± 0.6 ^(R)^
		9	9	9	9
3	*A. flavus (AM3)*	2.3 ± 1.3	7.7 ± 0.5	8.7 ± 0.5	10.7 ± 0.5 ^(R)^
		10.0	10.0	10.0	10.0
4	*A. flavus (AM4)*	2 ± 1.2	6.3 ± 0.5	9 ± 0	9.79 ± 0.5 ^(R)^
		8	8	8	8
5	*A. flavus (AM5)*	9.3 ± 0.6	8.7 ± 0.6	9 ± 0	9 ± 0 ^(R)^
		10	10	10	10
6	*A. fumigatus (AM6)*	7.7 ± 0.6	10.7 ± 0.6	11.7 ± 0.6	19.0 ± 1 ^(R)^
		10	10	10	10
7	*A. fumigatus (AM7)*	7.7 ± 0.6	10.7 ± 0.6	12.3 ± 0.6	18.7 ± 0.6 ^(R)^
		10	10	10	10
8	*A. niger (AM8)*	0 ± 0.6	0 ± 0.6	6.7 ± 0.6	12.7 ± 0.6 ^(R)^
		0	0	0	0
9	*A. niger (AM9)*	2 ± 3.5	6.7 ± 0.6	9 ± 2	18.7 ± 0.6 ^(R)^
		7	7	7	7
10	*A. terreus (AM10)*	6.7 ± 0.6	7.7 ± 0.6	8.7 ± 0.6	14.7 ± 0.6 ^(R)^
		9	9	9	9
11	*A. flavus (AM11)*	5.7 ± 0.6	7.7 ± 0.6	9.7 ± 0.6	10.7 ± 0.6 ^(R)^
		9	9	9	9
12	*A. flavus (AM12)*	2 ± 0.6	6.7 ± 0.6	7.3 ± 0.6	9.7± 0.6 ^(R)^
		8	8	8	8
13	*A. flavus (AM13)*	0 ± 0	0 ± 0	6.7 ± 0.6	9.3 ± 0.6 ^(R)^
		0	0	0	0
14	*A. terreus* 8	7 ± 0	6.7 ± 0.6	7.7 ± 0.6	11.7 ± 0.6 ^(R)^
		10	10	10	10
15	*A. flavus (AM14)*	7.7 ± 0.6	8 ± 0	9 ± 0	12.3 ± 0.6 ^(R)^
		9	9	9	9
16	*A. flavus (AM15)*	0 ± 0	0 ± 0	0 ± 0	0 ± 0 ^(R)^
		0	0	0	0
17	*A. flavus (AM16)*	6.7 ± 0.6	7.3 ± 1.2	8.7 ± 0.6	9.7 ± 0.6 ^(R)^
		7	7	7	7
18	*A. niger (AM17)*	0 ± 0	6 ± 0	12.7 ± 0.6	16.7 ± 0.6 ^(S)^
		0	0	0	0

R: Resistant, S: Sensitive.

**Table 3:** Susceptibility of fungal species to different concentrations of Voriconazole (disc method).

N	Isolate	Inhibition Zone (mm)			
		1 µg/mL	2 µg/mL	4 µg/mL	10 µg/mL
1	*A. flavus (AM1)*	0 ± 0	6.3 ± 0.6	6.7 ± 0.6	7 ± 0 ^(R)^
		7	7	7	7
2	*A. flavus (AM2)*	0 ± 0	6 ± 0	6.3 ± 0.6	6.3 ± 0.6 ^(R)^
		6	6	6	6
3	*A. flavus (AM3)*	0 ± 0	0 ± 0	0 ± 0	6.0 ± 0 ^(R)^
		0	0	0	0
4	*A. flavus (AM4)*	0 ± 0	0 ± 0	0 ± 0	6.7 ± 0.6 ^(R)^
		0	0	0	0
5	*A. flavus (AM5)*	6 ± 0	6.7 ± 0.6	6.7 ± 0.6	8.7 ± 0.6 ^(R)^
		8	8	8	8
6	*A. fumigatus (AM6)*	0 ± 0	0 ± 0	7.7 ± 0.6	10.3 ± 0.6 ^(R)^
		0	0	0	0
7	*A. fumigatus (AM7)*	0 ± 0	0 ± 0	0 ± 0	9 ± 0 ^(R)^
		0	0	0	0
8	*A. niger (AM8)*	0 ± 0	0 ± 0	0 ± 0	8 ± 0 ^(R)^
		0	0	0	0
9	*A. niger (AM9)*	0 ± 0	6 ± 0	7.7 ± 0.6	13.3 ± 0.6 ^(R)^
		0	0	0	0
10	*A. terreus (AM10)*	6 ± 0	6 ± 0	6.7 ± 0.6	7 ± 0 ^(R)^
		7	7	7	7
11	*A. flavus (AM11)*	6 ± 0	6 ± 0	6.3 ± 0.6	7 ± 0 ^(R)^
		7	7	7	7
12	*A. flavus (AM12)*	0 ± 0	0 ± 0	0 ± 0	7.7 ± 0.6 ^(R)^
		7	7	7	7
13	*A. flavus (AM13)*	0 ± 0	0 ± 0	6.3 ± 0.6	7 ± 0.6 ^(R)^
		7	7	7	7
14	*A. terreus 8*	0 ± 0	0 ± 0	0 ± 0	0 ± 0 ^(R)^
		20	20	20	20
15	*A. flavus (AM14)*	0.0 ± 0	0.0 ± 0	0.0 ± 0	14.7 ± 0.6 ^(R)^
		0	0	0	0
16	*A. flavus (AM15)*	6.7 ± 0.6	7 ± 0	8.7 ± 0.6	9.7 ± 0.6 ^(R)^
		9	9	9	9
17	*A. flavus (AM16)*	6.7 ± 0.6	7 ± 0	8 ± 0	8.7 ± 0.6 ^(R)^
		8	8	8	8
18	*A. niger (AM17)*	0 ± 0	0 ± 0	6.7 ± 0.6	16.7 ± 0.6 ^(R)^
		0	0	0	0

R: Resistant.

**Table 4:** Susceptibility of fungal species to different concentrations of Micafungin.

N	Isolate	Inhibition Zone (mm)			
		1 µg/mL	2 µg/mL	4 µg/mL	10 µg/mL
1	*A. flavus (AM1)*	0 ± 0	0 ± 0	0 ± 0	6.3 ± 0.6 ^(R)^
		0	0	0	0
2	*A. flavus (AM2)*	0 ± 0	0 ± 0	0 ± 0	0 ± 0 ^(R)^
		0	0	0	0
3	*A. flavus (AM3)*	0 ± 0	0 ± 0	0 ± 0	0 ± 0 ^(R)^
		2	2	2	2
4	*A. flavus (AM4)*	0 ± 0	0 ± 0	0 ± 0	7 ± 0 ^(R)^
		0	0	0	0
5	*A. flavus (AM5)*	0 ± 0	0 ± 0	0 ± 0	6.7 ± 0.6 ^(R)^
		0	0	0	6.6
6	*A. fumigatus (AM6)*	2 ± 3.5	4 ± 3.5	6.7 ± 0.6	8 ± 1.7 ^(R)^
		0	0	0	0
7	*A. fumigatus (AM7)*	6 ± 0	6.3 ± 0.6	6.7 ± 0.6	7.7 ± 1.5 ^(R)^
		0	0	0	0
8	*A. niger (AM8)*	0 ± 0	0 ± 0	0 ± 0	3.3 ± 5.8 ^(R)^
		0	0	0	0
9	*A. niger (AM9)*	0 ± 0	6.7 ± 0.6	6.7 ± 0.6	9.7 ± 0.6 ^(R)^
		11	11	11	11
10	*A. terreus (AM10)*	0 ± 0	0 ± 0	0 ± 0	0 ± 0 ^(R)^
		9	9	9	9
11	*A. flavus (AM11)*	0 ± 0	0 ± 0	0 ± 0	0 ± 0 ^(R)^
		0	0	0	0
12	*A. flavus (AM12)*	0 ± 0	0 ± 0	6.3 ± 0.6	5 ± 4.4 ^(R)^
		0	0	0	0
13	*A. flavus (AM13)*	0 ± 0	0 ± 0	0 ± 0.6	7.3 ± 1.5 ^(R)^
		0	0	0	0
14	*A. terreus 8*	0 ± 0	0 ± 0	0 ± 0	9.7 ± 0.6 ^(R)^
		0	0	0	0
15	*A. flavus (AM14)*	0 ± 0	0 ± 0	0 ± 0	6.3 ± 0.6 ^(R)^
		0	0	0	0
16	*A. flavus (AM15)*	0 ± 0	0 ± 0	0 ± 0	0 ± 0 ^(R)^
		4.3	4.3	4.3	4.3
17	*A. flavus (AM16)*	0 ± 0	0 ± 0	0 ± 0	6.3 ± 0.6 ^(R)^
		0	0	0	0
18	*A. niger (AM17)*	0 ± 0	0 ± 0	0 ± 0	0 ± 0 ^(R)^
		4.3	4.3	4.3	4.3

R: Resistant.

**Table 5:** Susceptibility of fungal species to different concentrations of Amphotericin B (broth microdilution method).

Isolate	Growth Rate/Score				
	1 µg/mL	2 µg/mL	4 µg/mL	8 µg/mL	16 µg/mL
*A. flavus (AM1)*	3 ± 0.6	2 ± 0	1 ± 0	0± 0 ^(R)^	0 ± 0
	4	4	4	4	4
*A. flavus (AM2)*	3 ± 0	1 ± 0	0 ± 0 ^(R)^	0 ± 0	0 ± 0
	4	4	4	4	4
*A. flavus (AM3)*	4 ± 0	3 ± 0.6	2 ± 1.2	1 ± 1.2	1 ± 0.6 ^(R)^
	4	4	4	4	4
*A. flavus (AM4)*	4 ± 0	4 ± 0	4 ± 0.6	2 ± 1.7	0 ± 0 ^(R)^
	4	4	4	4	4
*A. flavus (AM5)*	4 ± 0	4 ± 0	3 ± 1.2	1 ± 0	0 ± 0 ^(R)^
	4	4	4	4	4
*A. fumigatus (AM6)*	4 ± 0	4 ± 0.6	2 ± 1.2	1 ± 0	0 ± 0.6 ^(R)^
	4	4	4	4	4
*A. fumigatus (AM7)*	4 ± 0	3 ± 0.6	3 ± 2.3	0 ± 0 ^(R)^	0 ± 0
	4	4	4	4	4
*A. niger (AM8)*	4 ± 0	3 ± 2.3	1 ± 1.2	1 ± 0.6	0 ± 0 ^(R)^
	4	4	4	4	4
*A. niger (AM9)*	4 ± 0	4 ± 0	0 ± 0 ^(R)^	0 ± 0	0 ± 0
	4	4	4	4	4
*A. terreus (AM10)*	4 ± 0	4 ± 0	4 ± 0	4 ± 0	3 ± 1.2 ^(R)^
	4	4	4	4	4
*A. flavus (AM11)*	4 ± 0	4 ± 0	4 ± 0	3 ± 0	2 ± 0 ^(R)^
	4	4	4	4	4
*A. flavus (AM12)*	4 ± 1	3 ± 1.2	3 ± 1.7	1 ± 1.5	0 ± 0 ^(R)^
	4	4	4	4	4
*A. flavus (AM13)*	4 ± 0	4 ± 0	3 ± 0.6	0 ± 0.6 ^(R)^	0 ± 0
	4	4	4	4	4
*A. terreus 8*	4 ± 0	4 ± 0	4 ± 0	4 ± 0.6	2 ± 0.6^(R)^
	4	4	4	4	4
*A. flavus (AM14)*	4 ± 0	3 ± 0	2 ± 0	0 ± 0 ^(R)^	0 ± 0
	4	4	4	4	4
*A. flavus (AM15)*	4 ± 0	3 ± 1.2	3 ± 2.3	3 ± 2.3	0 ± 0 ^(R)^
	4	4	4	4	4
*A. flavus (AM16)*	4 ± 0	2 ± 0	0 ± 0 ^(R)^	0 ± 0	0 ± 0
	4	4	4	4	4
*A. niger (AM17)*	2 ± 0	2 ± 0	1 ± 0	0 ± 0 ^(R)^	0 ± 0
	4	4	4	4	4

R: Resistant.

**Table 6:** Susceptibility of fungal species to different concentrations of Voriconazole (broth microdilution method).

Isolate	Growth Rate/Score				
	1 µg/mL	2 µg/mL	4 µg/mL	8 µg/mL	16 µg/mL
*A. flavus (AM1)*	4 ± 0	3 ± 0	3 ± 0	2 ± 0	2 ± 0 ^(R)^
	4	4	4	4	4
*A. flavus (AM2)*	4 ± 0	4 ± 0	4 ± 0.6	3 ± 0	1 ± 0 ^(R)^
	4	4	4	4	4
*A. flavus (AM3)*	4 ± 0	3 ± 0	2 ± 0	2 ± 0	1 ± 0 ^(R)^
	4	4	4	4	4
*A. flavus (AM4)*	4 ± 0	3 ± 0	3 ± 0	3 ± 0	3 ± 0 ^(R)^
	4	4	4	4	4
*A. flavus (AM5)*	3 ± 0	3 ± 0	2 ± 0	1 ± 0	0 ± 0 ^(R)^
	4	4	4	4	4
*A. fumigatus (AM6)*	2 ± 0	0 ± 0 ^(I)^	0 ± 0	0 ± 0	0 ± 0
	4	4	4	4	4
*A. fumigatus (AM7)*	3 ± 0	0 ± 0 ^(I)^	0 ± 0	0 ± 0	0 ± 0
	4	4	4	4	4
*A. niger (AM8)*	2 ± 0	2 ± 0	2 ± 0	1 ± 0	1 ± 0 ^(R)^
	4	4	4	4	4
*A. niger (AM9)*	0 ± 0 ^(S)^	0 ± 0	0 ± 0	0 ± 0	0 ± 0
	4	4	4	4	4
*A. terreus (AM10)*	3 ± 0	3 ± 0	2 ± 0	1 ± 0	1 ± 0 ^(R)^
	4	4	4	4	4
*A. flavus (AM11)*	2 ± 0	2 ± 0	2 ± 0	2 ± 0	0 ± 0 ^(R)^
	4	4	4	4	4
*A. flavus (AM12)*	3 ± 0	3 ± 0	2 ± 0	1 ± 0	0 ± 0 ^(R)^
	4	4	4	4	4
*A. flavus (AM13)*	3 ± 0	3 ± 0	2 ± 0	2 ± 0	1 ± 0 ^(R)^
	4	4	4	4	4
*A. terreus 8*	3 ± 0	3 ± 0	3 ± 0	3 ± 0	1 ± 0.6 ^(R)^
	4	4	4	4	4
*A. flavus (AM14)*	3 ± 0	3 ± 0	3 ± 0	3 ± 0	2 ± 0 ^(R)^
	4	4	4	4	4
*A. flavus (AM15)*	4 ± 0	4 ± 0	4 ± 0	3 ± 0	2 ± 0 ^(R)^
	4	4	4	4	4
*A. flavus (AM16)*	0 ± 0 ^(S)^	0 ± 0	0 ± 0	0 ± 0	0 ± 0
	4	4	4	4	4
*A. niger (AM17)*	2 ± 0	0 ± 0 ^(I)^	0 ± 0	0 ± 0	0 ± 0
	4	4	4	4	4

R: Resistant, I: Intermediate, and S: Sensitive.

**Table 7:** Susceptibility of fungal species to different concentrations of Micafungin (broth microdilution method).

Isolate	Growth Rate/Score				
	1 µg/mL	2 µg/mL	4 µg/mL	8 µg/mL	16 µg/mL
*A. flavus (AM1)*	4 ± 0	4 ± 0	4 ± 0	3 ± 0	1 ± 0 ^(R)^
	4	4	4	4	4
*A. flavus (AM2)*	4 ± 0	4 ± 0	4 ± 0	4 ± 0	0 ± 0 ^(R)^
	4	4	4	4	4
*A. flavus (AM3)*	4 ± 0	4 ± 0	4 ± 0	4 ± 0	3 ± 0 ^(R)^
	4	4	4	4	4
*A. flavus (AM4)*	4 ± 0	4 ± 0	4 ± 0	4 ± 0	2 ± 0 ^(R)^
	4	4	4	4	4
*A. flavus (AM5)*	4 ± 0	4 ± 0	4 ± 0	4 ± 0	1 ± 0 ^(R)^
	4	4	4	4	4
*A. fumigatus (AM6)*	4 ± 0	4 ± 0	4 ± 0	4 ± 0	3 ± 0 ^(R)^
	4	4	4	4	4
*A. fumigatus (AM7)*	4 ± 0	4 ± 0	4 ± 0	4 ± 0	3 ± 0 ^(R)^
	4	4	4	4	4
*A. niger (AM8)*	4 ± 0	3 ± 0	2 ± 0	1 ± 0	1 ± 0 ^(R)^
	4	4	4	4	4
*A. niger (AM9)*	4 ± 0	3 ± 0	2 ± 0	1 ± 0	1 ± 0 ^(R)^
	4	4	4	4	4
*A. terreus (AM10)*	4 ± 0	4 ± 0	4 ± 0	4 ± 0	2 ± 0 ^(R)^
	4	4	4	4	4
*A. flavus (AM11)*	4 ± 0	4 ± 0	4 ± 0	3 ± 0	1 ± 0 ^(R)^
	4	4	4	4	4
*A. flavus (AM12)*	4 ± 0	4 ± 0	4 ± 0	3 ± 0	1 ± 0 ^(R)^
	4	4	4	4	4
*A. flavus (AM13)*	4 ± 0	4 ± 0	3 ± 0	2 ± 0	1 ± 0 ^(R)^
	4	4	4	4	4
*A. terreus 8*	4 ± 0	4 ± 0	4 ± 0	4 ± 0	3 ± 0 ^(R)^
	4	4	4	4	4
*A. flavus (AM14)*	4 ± 0	4 ± 0	4 ± 0	4 ± 0	1 ± 0 ^(R)^
	4	4	4	4	4
*A. flavus (AM15)*	4 ± 0	4 ± 0	4 ± 0	4 ± 0	3 ± 0 ^(R)^
	4	4	4	4	4
*A. flavus (AM16)*	3 ± 0	3 ± 0	2 ± 0.6	1 ± 0	0 ± 0 ^(R)^
	4	4	4	4	4
*A. niger (AM17)*	0 ± 0 ^(S)^	0 ± 0	0 ± 0	0 ± 0	0 ± 0
	4	4	4	4	4

R: Resistant, S: Sensitive.

**Table 8:** Efficacy of different concentrations of AuNPs against fungi as the zone of inhibition in disc method.

No.	Fungi	Inhibition Zone (mm)				
		25	50	100	200	1000
1	*A. flavus (AM1)*	0 ± 0	0 ± 0	0 ± 0	0 ± 0	0 ± 0
2	*A. flavus (AM2)*	0 ± 0	6.7 ± 0.6	7 ± 1	7.3 ± 0.6	9.3 ± 2.3
3	*A. flavus (AM3)*	0 ± 0	0 ± 0	0 ± 0	0 ± 0	0 ± 0
4	*A. flavus (AM4)*	0 ± 0	0 ± 0	0 ± 0	0 ± 0	0 ± 0
5	*A. flavus (AM5)*	0 ± 0	0 ± 0	0 ± 0	0 ± 0	0 ± 0
6	*A. fumigatus (AM6)*	0 ± 0	0 ± 0	0 ± 0	0 ± 0	0 ± 0
7	*A. fumigatus (AM7)*	0 ± 0	0 ± 0	0 ± 0	0 ± 0	0 ± 0
8	*A. niger (AM8)*	0 ± 0	0 ± 0	0 ± 0	0 ± 0	0 ± 0
9	*A. niger (AM9)*	0 ± 0	0 ± 0	0 ± 0	0 ± 0	0 ± 0
10	*A. terreus (AM10)*	6.7 ± 0.6	7 ± 0	7 ± 0	7 ± 0	7 ± 0
11	*A. flavus (AM11)*	0 ± 0	6.7 ± 0.6	6.7 ± 0.6	7 ± 0	8.7 ± 0.6
12	*A. flavus (AM12)*	0 ± 0	0 ± 0	0 ± 0	0 ± 0	0 ± 0
13	*A. flavus (AM13)*	0 ± 0	0 ± 0	0 ± 0	0 ± 0	0 ± 0
14	*A. terreus 8*	0 ± 0	0 ± 0	0 ± 0	0 ± 0	6.3 ± 0.6
15	*A. flavus (AM14)*	0 ± 0	0 ± 0	0 ± 0	0 ± 0	0 ± 0
16	*A. flavus (AM15)*	0 ± 0	0 ± 0	0 ± 0	0 ± 0	6.7 ± 0.6
17	*A. flavus (AM16)*	0 ± 0	0 ± 0	0 ± 0	0 ± 0	0 ± 0
18	*A. niger (AM17)*	0 ± 0	0 ± 0	0 ± 0	0 ± 0	0 ± 0

**Table 9:** Effect of different concentrations of AuNPs on fungal growth (growth rate score 0 = no growth, 4 = max growth) measured using broth microdilution technique.

No.	Fungi	Growth Rate (Scores/4)				
		25	50	100	200	1000
1	*A. flavus (AM2)*	3 ± 0 *	3 ± 0	3 ± 0	3 ± 0	2 ± 0
2	*A. terreus (AM10)*	4 ± 0	4 ± 0	4 ± 0	2 ± 0 *	2 ± 0
3	*A. flavus (AM11)*	4 ± 0	4 ± 0	4 ± 0	3 ± 0 *	1 ± 0
4	*A. terreus 8*	4 ± 0	4 ± 0	4 ± 0	3 ± 0 *	2 ± 0
5	*A. flavus (AM15)*	4 ± 0	3 ± 0 *	1 ± 0	1 ± 0	0 ± 0 **

*: MEC, **: MIC.

## Data Availability

Not applicable.
